# Supplementary material for: CLARITY-compatible lipophilic dyes for electrode marking and neuronal tracing
Source: Sci Rep. 2016 Sep 6;6:32674. doi: 10.1038/srep32674 (PMC5011694; doi:10.1038/srep32674)
Supplement: Supplementary Information [file srep32674-s1.pdf]

Supplementary material for:

CLARITY-compatible lipophilic dyes for electrode marking and neuronal tracing

by Kristian H. R. Jensen[1] and Rune W. Berg[1]*

[1]University of Copenhagen, Department of Neuroscience and Pharmacology, Copenhagen, DK-2200, Denmark
*runeb@sund.ku.dk

Includes 4 figures and 1 table.

Supplementary figure 1

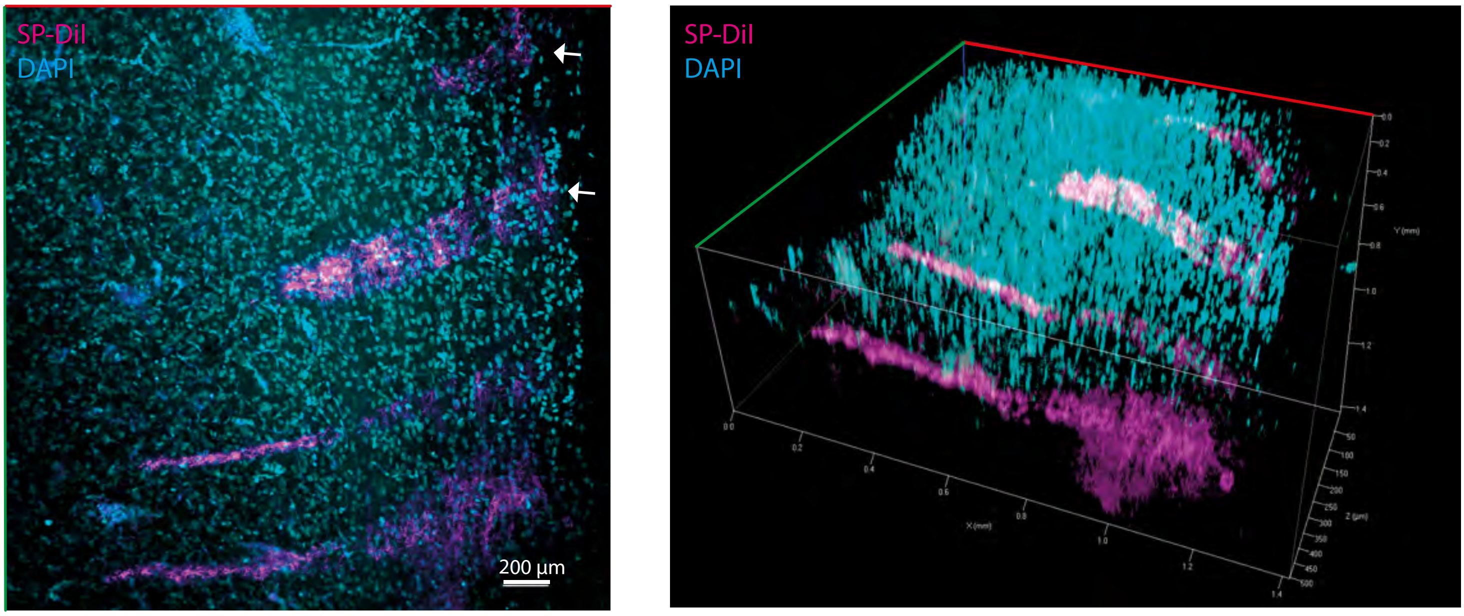

Post-mortem SP-DiI tracing in pre-fixed rat spinal cord treated with CLARITY.
(a) Sagittal view of maximum intensity projection and (b) 3D-projection of the electrode trace (magenta) with DAPI (cyan) after CLARITY. (c) Macroscopic image of a non-cleared (left) and a CLARITY-cleared (right) lumbar section of the rat spinal cord. (d) Widefield image, ventral view, of the CLARITY treated rat spinal cord with injection sites of a 4 shank multielectrode with SP-DiI fluorescence (magenta).The tissue was pre-fixed in PFA and the insertion time was 30 min. Lipid clearing in CLARITY took 6 days. We used a damaged multielectrode where two of the shanks were broken to shorter lengths (arrows). (a, b) 104 confocal images, Z = 530 µm. (c) was imaged in a petri dish with 80% glycerol in PBS and the background squares are 5x5 mm.

c

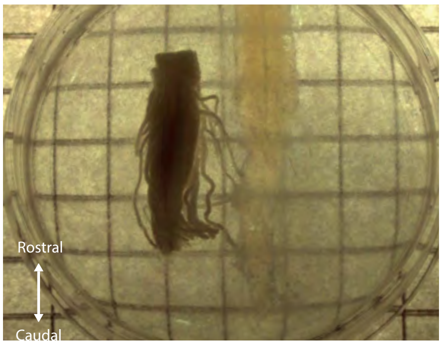

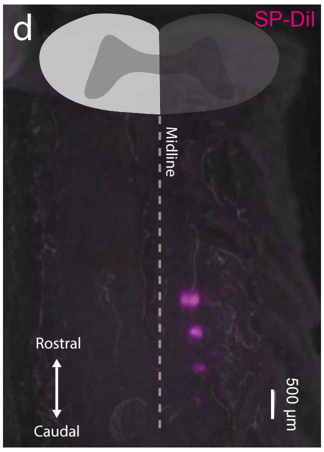

Supplementary figure 2

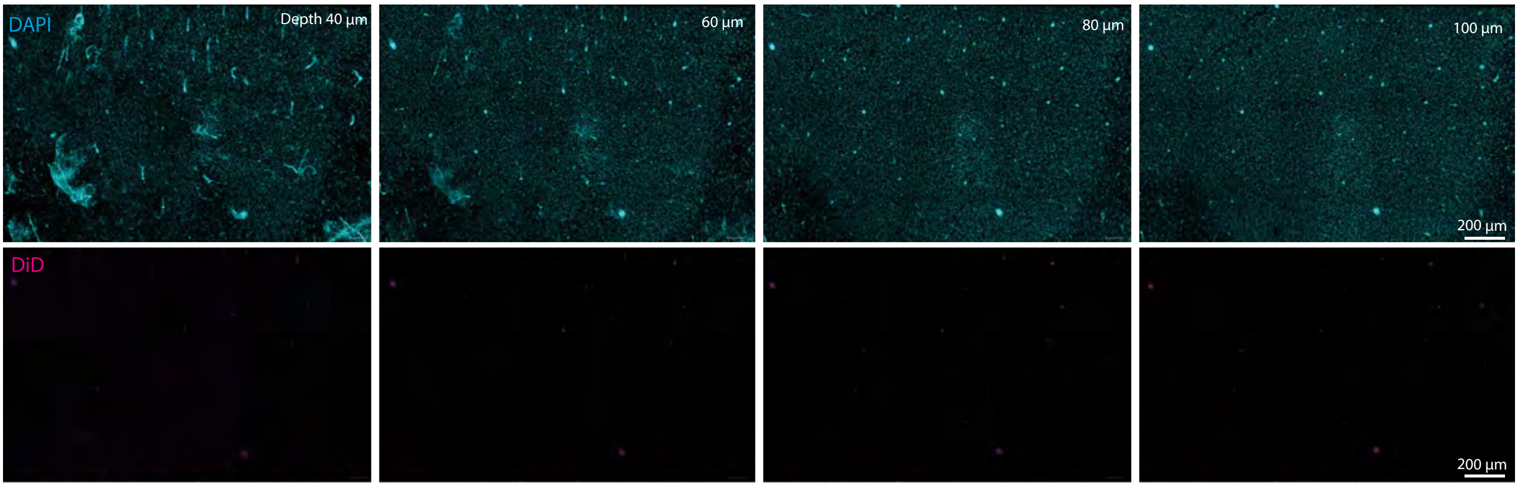

Non-fixable lipophilic dye washed out during CLARITY treatment.
Confocal images of a 1 mm sagittal slice of the mouse auditory cortex treated with CLARITY and stained with DAPI (cyan, top). A multielectrode (4 shank, 125 µm apart) dipped in a solution containing DiD (a redshifted variant of DiI) was inserted in the anaesthetized animal. This gives reliable tracing in conventional histology. However, in CLARITY the DiD electrode trace (magenta, bottom) could not be seen in the eyepiece or during image capture at any focus depth, even though the 5 mW, 633 nm laser was at 20%. The dots observed in the DiD correspond to autofluorescence from blood vessel also seen in the DAPI stain. This confirms that non-fixable lipophilic dyes are incompatible with lipid-clearing. Similar results were seen with Sca/eA2 in our lab (not shown). Images are 4.08 x 2.58 mm (15 images overlapping 5%) with non-overlapping 20 µm optical sections taken at a depth range of 0-120 µm measured just below the cortex surface. Depths 40, 60, 80, and 100 µm are shown here.

Supplementary figure 3

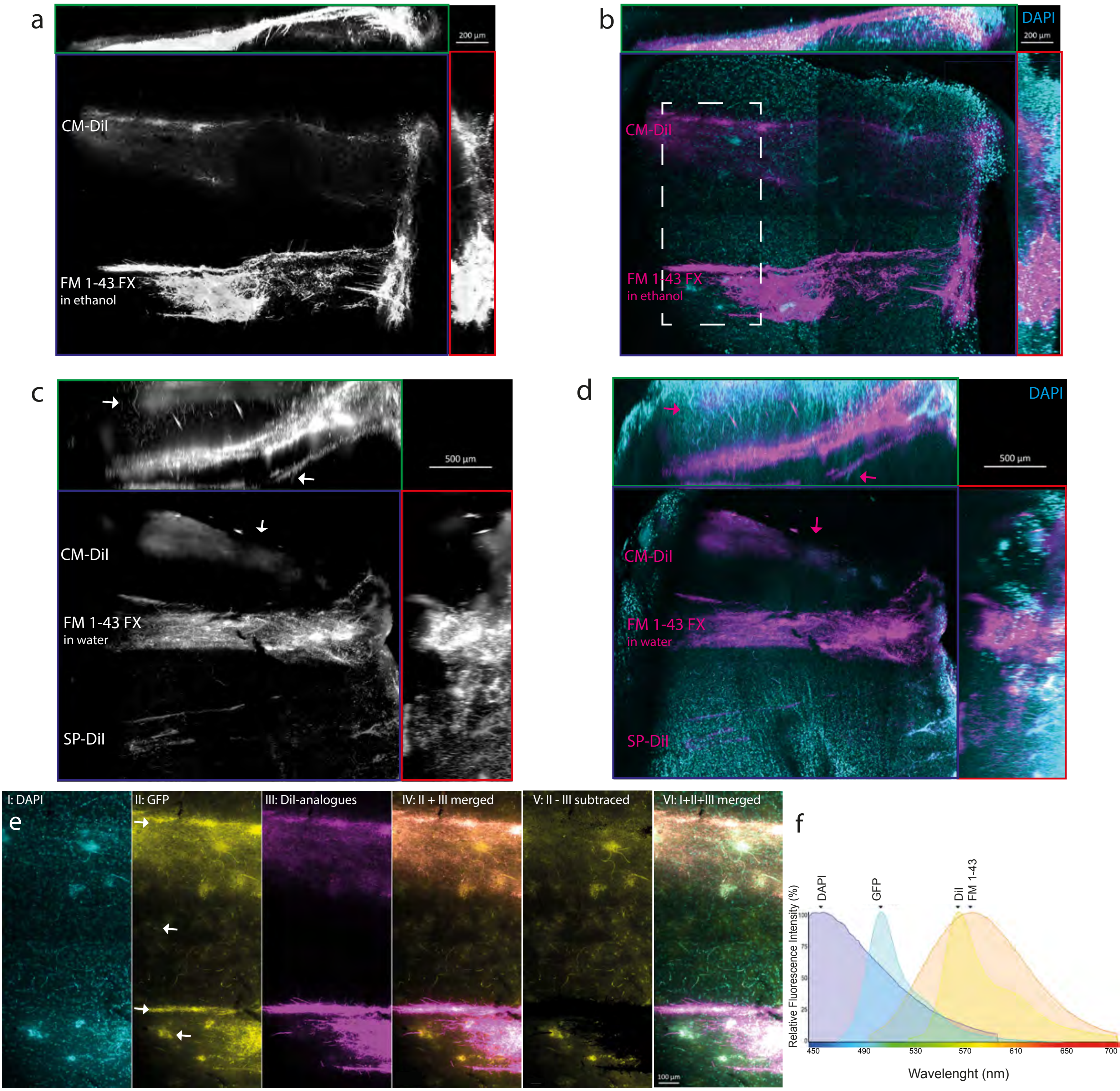

Imaging DiI-analogues with GFP-expressing astrocytes shows spectral cross-talk.
Sagittal view of GFP-expressing mouse spinal cord. White traces are from a multielectrode with four shanks dipped in either CM-DiI dissolved in ethanol (a) FM 1-43FX in ethanol (a) or water (c), or SP-DiI in ethanol (c). Magenta traces in images (b) and (d) are (a) and (c), respectively, with a DAPI counterstain (cyan). Exposure time was 8 min and on the contralateral side of the spinal cord of the animal: (a, b) from the left side and (c, d) from the right side. The right side SP-DiI electrode insertion was the last shortly before the respiratory arrest of the animal. Notice the CM-DiI trace is located superficially (right-facing arrow) and close to the tissue cut end (downward arrow), which may contribute to the diffuse staining. Dissolving FM 1-43FX in ethanol (a) versus water (c) did not seem to make a difference in the degree of staining. The depth of the SP-DiI trace (left-facing arrow) contributes to the limited fluorescence captured in the image. (e) Closeup of the dashed box from (b) showing crosstalk between dyes. The panels include images (II) of the GFP-expressing astrocytes (left-facing arrows) not shown in the above images (a-d). Spectral crosstalk between GFP and the DiI is seen in (III, right-facing arrows), which was reduced by simple subtraction of the image of the DiI-analogues from the GFP image (V). The challenge in multi-channel imaging where both FM 1-43FX and DAPI have broad emission spectra overlapping with GFP (f) is evident in the merged images of all fluorophores (VI). (f) The emission spectra of DAPI, GFP, DiI and FM 1-43 in the 450-700 nm range (SpectraViewer, from the website of Thermo Fisher Science). All images are maximum intensity projections of Z(blue box), X(red box) and Y(green box) planes of the (a, b) 56 confocal image stack (2x2 tiles, Z = 269 µm) and (c, d) 125 confocal image stack (2x2 tiles, Z = 866 µm). The output of the exciting laser is the same for all tiles; there is a slight light fall-off at the tile edges.

Supplementary figure 4

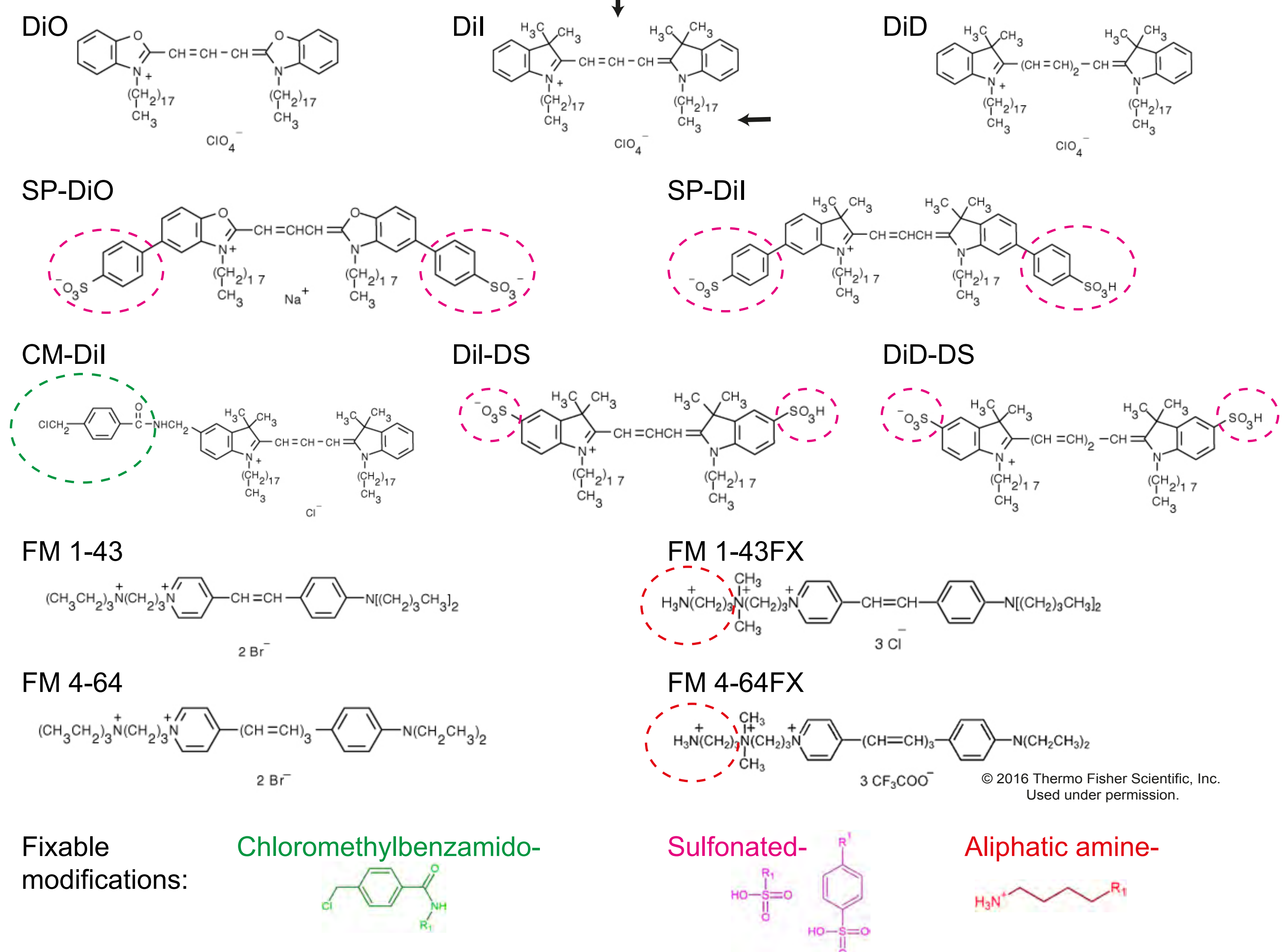

Chemical structures of available DiI and FM dyes.
Modifications that ensure fixability (broken circles) shown in colours that indicate their modification. The derivatives of DiI with differing carbon bridge lengths between the indoline rings (vertical arrow) have different excitation and emission wavelengths e.g. DiD red-shifted version with a 5 rather than 3 carbon bridge. A substitute of carbon to oxygen in the indoline rings moves DiO to lower wavelengths. The lipophilic property is dependent on akyl-chain lengths (horizontal arrow). FM 1-43 has a red-shifted variant, FM 4-64, with a 6 rather than 2 carbon bridge between the aromatic rings.

The images are the copyrighted property owned by Life Technologies Corporation, a part of Thermo Fisher Scientific Inc. www.thermofisher.com

Supplementary table 1

Overview of commercially available DiI and FM dyes†.
The dyes are grouped by type and modification. 1) FM 1-43 Ex = 479 nm, Em = 598 nm when bound to phospholipid bilayer membranes and Em= 565 nm when bound to synaptosomal membranes. 2) Ex, Em and ε determined for dye bound to detergent micelles (20 mg/ml CHAPS in H_2O). These dyes are essentially nonfluorescent in pure water. *Spectral properties determined in methanol unless noted below. ε: Molar attenuation coefficient. †Modified from Molecular Probes Handbook (Thermo Science Fisher). Ex: excitation. Em: Emission.

Trival name	Name	Fixable Modification	Molar mass g/mol	Excitation* nm	Emisson* nm	Stokes shift nm	ε* m^2/mol
DiO	DiOC18(3)		881.72	484	501	17	154,000
DiI	DiIC18(3)		933.88	549	565	16	148,000
DiD	DiIC18(5)		1052.08	644	663	19	193,000
SP-DiO	SP-DiOC18(3)	sulfophenyl	1115.55	497	513	16	175,000
DiI-DS	DiIC18(3)-DS	sulfonate	993.54	555	570	15	144,000
SP-DiI	SP-DiIC18(3)	sulfophenyl	1145.73	556	573	17	164,000
DiD-DS	DiIC18(5)-DS	sulfonate	1019.58	650	670	20	247,000
CM-DiI	CellTracker™ CM-DiI	chloromethyl	1051.50	553	570	17	134,000
	FM® 1-43[1]		611.55	471	581	110	38,000
	FM® 4-64[2]		607.51	505	725	220	47,000
	FM® 1-43FX[1]	aliphatic amine	560.09	471	581	110	38,000
	FM® 4-64FX[2]	aliphatic amine	788.75	505	725	220	47,000
